# Bis(2,6-diamino­pyridinium) bis­(pyridine-2,6-dicarboxyl­ato)zincate(II) monohydrate

**DOI:** 10.1107/S1600536811018204

**Published:** 2011-05-20

**Authors:** Masoumeh Tabatabaee, Marjan Tahriri, Mozhgan Tahriri, Michal Dušek, Karla Fejfarová

**Affiliations:** a Department of Chemistry,Yazd Branch, Islamic Azad University, Yazd, Iran; b Institute of Physics ASCR, v.v.i., Na Slovance 2, 182 21 Praha 8, Czech Republic

## Abstract

In the title hydrated mol­ecular salt, (C_5_H_8_N_3_)_2_[Zn(C_7_H_3_NO_4_)_2_]·H_2_O, the Zn^II^ atom is coordinated by two *O*,*N*,*O*′-tridentate pyridine-2,6-dicarboxyl­ate dianions, generating a slightly distorted *trans*-ZnN_2_O_4_ octa­hedral coordination geometry for the metal ion. In the crystal, a network of O—H⋯O, N—H⋯O and C—H⋯O hydrogen bonds involving the cations, anions and water mol­ecules results in a three-dimensional network.

## Related literature

For isostructural mol­ecular salts with Co and Ni, see: Moghimi *et al.* (2002**a* ▶,b* ▶). For related sturctures, see: Tabatabaee *et al.* (2009 ▶); Ranjbar *et al.* (2002 ▶); Moghimi *et al.* (2005 ▶).
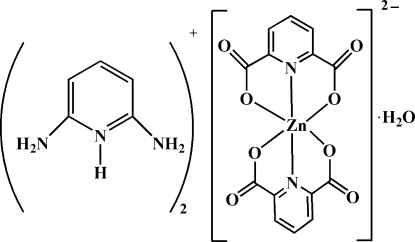


## Experimental

### 

#### Crystal data

(C_5_H_8_N_3_)_2_[Zn(C_7_H_3_NO_4_)_2_]·H_2_O
*M* *_r_* = 633.9
Monoclinic, 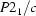

*a* = 8.2940 (3) Å
*b* = 13.2368 (4) Å
*c* = 23.8063 (7) Å
β = 104.995 (3)°
*V* = 2524.60 (14) Å^3^
*Z* = 4
Cu *K*α radiation
μ = 1.99 mm^−1^
*T* = 120 K
0.26 × 0.19 × 0.10 mm

#### Data collection

Oxford Diffraction Xcalibur diffractometer with an Atlas (Gemini ultra Cu) detector
Absorption correction: multi-scan (*CrysAlis PRO*; Oxford Diffraction, 2009 ▶) *T*_min_ = 0.142, *T*_max_ = 1
66673 measured reflections
4502 independent reflections
4175 reflections with *I* > 3σ(*I*)
*R*_int_ = 0.031

#### Refinement

*R*[*F* ^2^ > 2σ(*F* ^2^)] = 0.024
*wR*(*F* ^2^) = 0.077
*S* = 1.61
4502 reflections
415 parameters
12 restraints
H atoms treated by a mixture of independent and constrained refinement
Δρ_max_ = 0.20 e Å^−3^
Δρ_min_ = −0.32 e Å^−3^

### 

Data collection: *CrysAlis PRO* (Oxford Diffraction, 2009 ▶); cell refinement: *CrysAlis PRO*; data reduction: *CrysAlis PRO*; program(s) used to solve structure: *SIR2002* (Burla *et al.*, 2003 ▶); program(s) used to refine structure: *JANA2006* (Petříček *et al.*, 2007) ▶; molecular graphics: *DIAMOND* (Brandenburg & Putz, 2005 ▶); software used to prepare material for publication: *JANA2006* ▶.

## Supplementary Material

Crystal structure: contains datablocks global, I. DOI: 10.1107/S1600536811018204/hb5879sup1.cif

Structure factors: contains datablocks I. DOI: 10.1107/S1600536811018204/hb5879Isup2.hkl

Additional supplementary materials:  crystallographic information; 3D view; checkCIF report

## Figures and Tables

**Table 1:** Selected bond lengths (Å)

Zn1—O1	2.1725 (10)
Zn1—O3	2.1886 (11)
Zn1—O5	2.2595 (9)
Zn1—O7	2.2372 (8)
Zn1—N1	2.0107 (11)
Zn1—N2	2.0001 (11)

**Table 2:** Hydrogen-bond geometry (Å, °)

*D*—H⋯*A*	*D*—H	H⋯*A*	*D*⋯*A*	*D*—H⋯*A*
C10—H10⋯O3^i^	0.96	2.49	3.1769 (16)	129
C18—H18⋯O4	0.96	2.59	3.3556 (16)	137
C22—H22⋯O3^ii^	0.96	2.59	3.3789 (17)	139
N3—H3*n*⋯O8^ii^	0.854 (13)	1.948 (13)	2.7845 (13)	166.0 (16)
N4—H4*n*⋯O4^iii^	0.845 (17)	2.096 (17)	2.9287 (16)	168.4 (14)
N4—H4*m*⋯O7^ii^	0.838 (14)	2.357 (14)	3.1874 (14)	171.0 (17)
N5—H5*n*⋯O4	0.878 (15)	2.206 (14)	3.0600 (14)	164.2 (15)
N5—H5*m*⋯O9^iv^	0.872 (15)	2.066 (15)	2.9203 (15)	166.3 (17)
N6—H6*n*⋯O6^v^	0.884 (14)	1.763 (14)	2.6381 (13)	169.9 (15)
N7—H7*n*⋯O2^vi^	0.866 (15)	2.032 (15)	2.8818 (15)	166.8 (17)
N8—H8*n*⋯O2^vii^	0.840 (18)	2.085 (18)	2.9206 (17)	172.4 (17)
O9—H9*o*⋯O1^v^	0.83 (2)	2.06 (2)	2.8936 (14)	174 (2)
O9—H9*p*⋯O8^viii^	0.866 (19)	1.928 (19)	2.7462 (14)	157.1 (18)

Symmetry codes: (i) 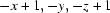; (ii) 
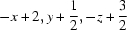
; (iii) 
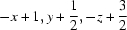
; (iv) 
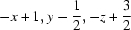
; (v) 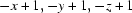; (vi) 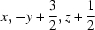; (vii) 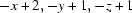; (viii) 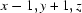.

**Fig. 1.**
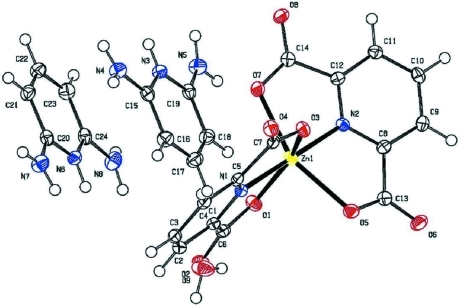
A view of (I), with displacement ellipsoids drawn at the 50% probability level.

**Fig. 2.**
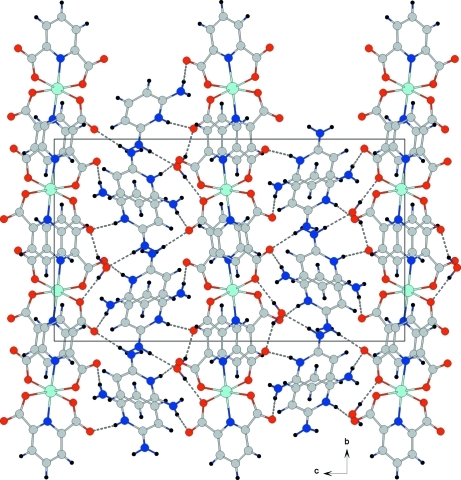
fragment of crystal packing. The strongest N—H···O and O—H···O interactions(with H···A distance up to 2.1 Å) interconnect all molecules of the structureinto three-dimensional network.

**Fig. 3.**
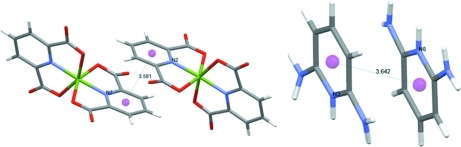
Representation π-π stacking between aromatic rings.

## supplementary crystallographic information

### Comment

The title compound, (I), consists of a [Zn(pydc)_2_]^2-^ dianion, two (pydaH)^+^ cations and one uncoordinated water molecule (Fig. 1) (pydcH_2_ = pyridine-2,6-dicarboxylic acid; pyda = 2,6-diaminopyridine), and is isostructural with Co (Moghimi *et al.* 2002*a*) and Ni (Moghimi *et al.* 2002*b*) compounds. Zn^II^ atom in the title compound is six-coordinated by two (pydc)^2–^ anions and the geometry of the resulting ZnN2O4 coordination can be described as distorted octahedral. The N atoms occupy the axial positions. The N1—Zn1—N2 angle (175.74 (5)°) deviates from linearity. The dihedral angle between the mean planes of the pyridine rings (A1 and A2, defined in Fig. 1) is 75.24 (6)°. Zn—N distances of (2.0011 (16) and 2.0107 (11) Å and Zn—O distances (Zn1—O1: 2.1725 (10), Zn1—O3: 2.1886 (11), Zn1—O5: 2.2595 (9) and Zn1—O7: 2.2372 (8) Å) are consistent with those found in (acrH)[Zn(pydc)(pydcH)].5H_2_O (Tabatabaee *et al.* 2009), (pydaH)[Zn(pydc)(pydcH)]. 3H_2_O (Ranjbar*et al.*. 2002) and(creatH)[Zn(pydc)(pydcH)]. 4H_2_O (Moghimi *et al.* 2005). There are some hydrogen bonding interactions such as O—H···O, N—H···O and C—H···O between cations, anions and uncoordinated water molecule (Fig 2, Table 1). As it is seen in Fig. 3, there are also π-π stacking interactions between the aromatic rings with centroid-centroiddistances of 3.5813 (8) Åfor *Cg*6···*Cg*6 (defined by atoms N2/C8 /C12, symmetry code: (2 - *x*,-*y*,1 - *z*) and 3.6421 (8) Å for *Cg*7···*Cg*8 (*Cg*7: N3/C15—C19 and *Cg*8:N6/ C20—C24, symmetry code: *x*, *y*, *z*).Ion pairing, hydrogen bonding, π–π stacking and van der Waals interactions are also effective for packingof the crystal structure. These interactions lead to formation of a three-dimensional supramolecular structure.

### Experimental

An aqueous solution of ZnSO_4_.7H_2_O, (0.288 g, 1 mmol) in water (10 ml) was added to a stirring solution of (20 ml)pyridine-2,6-dicarboxylic acid (0.167 g, 1 mmol) and 2,6-diaminopyridine (0.11 g, 1 mmol). The reaction mixture was stirred at 298 K for 3 h. Colorless blocks of (I) were obtained after few days at 277 K.

### Refinement

All hydrogen atoms were discernible in difference Fourier maps and could be refined to reasonable geometry. According to common practice H atoms attached to carbon atoms were kept in ideal positions with C–H distance 0.96 Å during the refinement. The position of hydrogen atoms of N atoms were refined with a restrictionon the N—H bond length 0.87 Å with σ of 0.02. The O–H distances were restrained to 0.84 Å with σ of 0.02. All H atoms were refined as riding with thermal displacement coefficients *U*_iso_(H) set to 1.5Ueq(O) for the water molecule and to to 1.2Ueq(C,*N*) for the CH–, NH– and NH2-groups.

### Crystal data

**Table:**

(C_5_H_8_N_3_)_2_[Zn(C_7_H_3_NO_4_)_2_]·H_2_O	*F*(000) = 1304
*M**_r_* = 633.9	*D*_x_ = 1.667 Mg m^−^^3^
Monoclinic, *P*2_1_/*c*	Cu *K*α radiation, λ = 1.5418 Å
Hall symbol: -P 2ybc	Cell parameters from 46751 reflections
*a* = 8.2940 (3) Å	θ = 3.3–67.0°
*b* = 13.2368 (4) Å	µ = 1.99 mm^−^^1^
*c* = 23.8063 (7) Å	*T* = 120 K
β = 104.995 (3)°	Block, colourless
*V* = 2524.60 (14) Å^3^	0.26 × 0.19 × 0.10 mm
*Z* = 4	

### Data collection

**Table:**

Oxford Diffraction Xcalibur diffractometer with an Atlas (Gemini ultra Cu) detector	4502 independent reflections
Radiation source: Enhance Ultra (Cu) X-ray Source	4175 reflections with *I* > 3σ(*I*)
mirror	*R*_int_ = 0.031
Detector resolution: 10.3784 pixels mm^-1^	θ_max_ = 67.1°, θ_min_ = 3.8°
Rotation method data acquisition using ω scans	*h* = −9→9
Absorption correction: multi-scan (*CrysAlis PRO*; Oxford Diffraction, 2009)	*k* = −15→15
*T*_min_ = 0.142, *T*_max_ = 1	*l* = −28→28
66673 measured reflections	

### Refinement

**Table:**

Refinement on *F*^2^	60 constraints
*R*[*F*^2^ > 2σ(*F*^2^)] = 0.024	H atoms treated by a mixture of independent and constrained refinement
*wR*(*F*^2^) = 0.077	Weighting scheme based on measured s.u.'s *w* = 1/[σ^2^(*I*) + 0.0016*I*^2^]
*S* = 1.61	(Δ/σ)_max_ = 0.002
4502 reflections	Δρ_max_ = 0.20 e Å^−^^3^
415 parameters	Δρ_min_ = −0.32 e Å^−^^3^
12 restraints	

### Special details

**Table:**

Experimental. CrysAlisPro, Oxford Diffraction (2009), Empirical absorption correction using spherical harmonics, implemented in SCALE3 ABSPACK scaling algorithm.
Refinement. The refinement was carried out against all reflections. The conventional *R*-factor is always based on *F*. The goodness of fit as well as the weighted *R*-factor are based on *F* and *F*^2^ for refinement carried out on *F* and *F*^2^, respectively. The threshold expression is used only for calculating *R*-factors *etc*. and it is not relevant to the choice of reflections for refinement.The program used for refinement, Jana2006, uses the weighting scheme based on the experimental expectations, see _refine_ls_weighting_details, that does not force *S* to be one. Therefore the values of *S* are usually larger than the ones from the *SHELX* program.

### Fractional atomic coordinates and isotropic or equivalent isotropic displacement parameters (Å^2^)

**Table:**

	*x*	*y*	*z*	*U*_iso_*/*U*_eq_	
Zn1	0.73417 (2)	0.251250 (11)	0.490469 (7)	0.01551 (8)	
O1	0.85536 (12)	0.30784 (7)	0.42606 (4)	0.0190 (3)	
O2	0.90680 (12)	0.45264 (7)	0.38497 (4)	0.0203 (3)	
O3	0.61404 (12)	0.27022 (7)	0.56153 (4)	0.0175 (3)	
O4	0.56144 (12)	0.38883 (7)	0.62210 (4)	0.0185 (3)	
O5	0.49822 (12)	0.22838 (7)	0.41879 (4)	0.0194 (3)	
O6	0.38232 (13)	0.10376 (7)	0.35720 (4)	0.0266 (3)	
O7	0.96377 (11)	0.19455 (7)	0.55438 (4)	0.0182 (3)	
O8	1.05526 (12)	0.05147 (7)	0.60233 (4)	0.0213 (3)	
N1	0.73813 (13)	0.40157 (8)	0.50291 (5)	0.0140 (3)	
N2	0.71778 (13)	0.10107 (8)	0.48158 (4)	0.0141 (3)	
N3	0.69954 (14)	0.60578 (8)	0.79837 (5)	0.0165 (3)	
N4	0.67598 (16)	0.73531 (9)	0.86105 (5)	0.0197 (4)	
N5	0.74582 (15)	0.47174 (9)	0.74142 (5)	0.0222 (4)	
N6	0.84303 (14)	0.80163 (8)	0.72445 (5)	0.0165 (3)	
N7	0.78444 (15)	0.93207 (9)	0.77987 (5)	0.0230 (4)	
N8	0.87253 (16)	0.67785 (9)	0.66049 (5)	0.0229 (4)	
C1	0.79561 (15)	0.46127 (9)	0.46716 (5)	0.0149 (4)	
C2	0.79764 (16)	0.56544 (10)	0.47355 (5)	0.0174 (4)	
C3	0.73738 (16)	0.60625 (10)	0.51793 (6)	0.0179 (4)	
C4	0.67565 (15)	0.54303 (10)	0.55434 (5)	0.0165 (4)	
C5	0.67867 (15)	0.44000 (9)	0.54554 (5)	0.0143 (4)	
C6	0.85813 (15)	0.40324 (10)	0.42179 (5)	0.0163 (4)	
C7	0.61279 (15)	0.35991 (9)	0.58001 (5)	0.0145 (4)	
C8	0.59296 (16)	0.06081 (9)	0.44044 (5)	0.0151 (4)	
C9	0.56638 (16)	−0.04242 (10)	0.43691 (5)	0.0170 (4)	
C10	0.66832 (16)	−0.10379 (10)	0.47856 (6)	0.0180 (4)	
C11	0.79717 (16)	−0.06116 (10)	0.52144 (6)	0.0169 (4)	
C12	0.82002 (16)	0.04249 (10)	0.52096 (5)	0.0145 (4)	
C13	0.48221 (16)	0.13793 (10)	0.40166 (5)	0.0173 (4)	
C14	0.95791 (16)	0.10108 (10)	0.56312 (5)	0.0162 (4)	
C15	0.62074 (16)	0.69111 (10)	0.80890 (5)	0.0169 (4)	
C16	0.48878 (17)	0.72880 (11)	0.76506 (6)	0.0204 (4)	
C17	0.44414 (17)	0.67788 (10)	0.71250 (6)	0.0222 (4)	
C18	0.52517 (17)	0.59131 (10)	0.70276 (6)	0.0200 (4)	
C19	0.65634 (16)	0.55427 (10)	0.74686 (5)	0.0170 (4)	
C20	0.88001 (16)	0.85182 (10)	0.77596 (5)	0.0170 (4)	
C21	1.01211 (17)	0.81712 (10)	0.82058 (6)	0.0197 (4)	
C22	1.09920 (18)	0.73294 (11)	0.81063 (6)	0.0219 (4)	
C23	1.05885 (17)	0.68247 (10)	0.75763 (6)	0.0214 (4)	
C24	0.92679 (17)	0.71923 (11)	0.71358 (6)	0.0179 (4)	
O9	0.13470 (16)	0.86262 (8)	0.64907 (5)	0.0345 (4)	
H2	0.839584	0.608225	0.447989	0.0209*	
H3	0.738256	0.678095	0.523516	0.0215*	
H4	0.632137	0.570423	0.584768	0.0198*	
H9	0.479364	−0.070943	0.406331	0.0204*	
H10	0.65006	−0.175446	0.477824	0.0216*	
H11	0.868503	−0.102603	0.550646	0.0203*	
H16	0.430228	0.788661	0.771181	0.0245*	
H17	0.354128	0.703644	0.681951	0.0266*	
H18	0.491551	0.557035	0.666037	0.024*	
H21	1.04204	0.850964	0.857491	0.0236*	
H22	1.190356	0.708568	0.841236	0.0263*	
H23	1.120325	0.623848	0.751528	0.0257*	
H3n	0.7811 (18)	0.5828 (12)	0.8249 (6)	0.0199*	
H4n	0.619 (2)	0.7832 (12)	0.8695 (7)	0.0237*	
H4m	0.7661 (19)	0.7184 (14)	0.8843 (7)	0.0237*	
H5n	0.711 (2)	0.4411 (13)	0.7078 (6)	0.0267*	
H5m	0.798 (2)	0.4391 (13)	0.7727 (7)	0.0267*	
H6n	0.7610 (18)	0.8265 (12)	0.6964 (6)	0.0198*	
H7n	0.805 (2)	0.9661 (13)	0.8120 (6)	0.0276*	
H7m	0.701 (2)	0.9456 (14)	0.7516 (7)	0.0276*	
H8n	0.933 (2)	0.6358 (12)	0.6492 (7)	0.0275*	
H8m	0.787 (2)	0.7089 (14)	0.6359 (7)	0.0275*	
H9o	0.130 (3)	0.8128 (14)	0.6273 (9)	0.0517*	
H9p	0.100 (3)	0.9126 (14)	0.6256 (9)	0.0517*	

### Atomic displacement parameters (Å^2^)

**Table:**

	*U*^11^	*U*^22^	*U*^33^	*U*^12^	*U*^13^	*U*^23^
Zn1	0.02022 (13)	0.00980 (13)	0.01659 (13)	−0.00032 (6)	0.00492 (9)	−0.00079 (5)
O1	0.0250 (5)	0.0150 (4)	0.0187 (4)	−0.0002 (4)	0.0087 (4)	−0.0008 (3)
O2	0.0226 (5)	0.0217 (5)	0.0178 (4)	0.0023 (4)	0.0075 (4)	0.0046 (4)
O3	0.0208 (5)	0.0134 (4)	0.0190 (5)	−0.0020 (4)	0.0066 (4)	−0.0013 (3)
O4	0.0212 (5)	0.0190 (5)	0.0163 (4)	−0.0019 (4)	0.0066 (4)	−0.0023 (3)
O5	0.0217 (5)	0.0151 (4)	0.0191 (5)	0.0023 (4)	0.0013 (4)	0.0006 (4)
O6	0.0279 (5)	0.0220 (5)	0.0223 (5)	0.0004 (4)	−0.0070 (4)	−0.0007 (4)
O7	0.0189 (5)	0.0142 (4)	0.0196 (4)	−0.0009 (3)	0.0018 (3)	−0.0017 (3)
O8	0.0203 (5)	0.0185 (5)	0.0204 (5)	0.0008 (4)	−0.0033 (4)	0.0003 (4)
N1	0.0143 (5)	0.0125 (6)	0.0141 (5)	−0.0004 (4)	0.0017 (4)	0.0007 (4)
N2	0.0156 (5)	0.0123 (5)	0.0146 (5)	0.0007 (4)	0.0044 (4)	0.0004 (4)
N3	0.0164 (6)	0.0170 (5)	0.0153 (5)	0.0016 (4)	0.0025 (4)	0.0005 (4)
N4	0.0209 (6)	0.0189 (5)	0.0190 (6)	0.0047 (5)	0.0044 (5)	−0.0023 (4)
N5	0.0288 (7)	0.0187 (6)	0.0184 (6)	0.0038 (5)	0.0045 (5)	−0.0021 (4)
N6	0.0173 (6)	0.0170 (5)	0.0142 (5)	−0.0004 (4)	0.0024 (4)	−0.0006 (4)
N7	0.0262 (7)	0.0201 (6)	0.0207 (6)	0.0027 (5)	0.0024 (5)	−0.0064 (5)
N8	0.0235 (7)	0.0246 (6)	0.0206 (6)	0.0001 (5)	0.0055 (5)	−0.0076 (5)
C1	0.0131 (6)	0.0156 (6)	0.0146 (6)	0.0002 (5)	0.0012 (5)	0.0022 (5)
C2	0.0165 (6)	0.0160 (6)	0.0179 (6)	−0.0003 (5)	0.0009 (5)	0.0037 (5)
C3	0.0180 (6)	0.0114 (6)	0.0210 (6)	0.0005 (5)	−0.0009 (5)	−0.0009 (5)
C4	0.0149 (6)	0.0166 (6)	0.0161 (6)	0.0006 (5)	0.0007 (5)	−0.0021 (5)
C5	0.0119 (6)	0.0162 (6)	0.0131 (6)	−0.0003 (5)	0.0000 (4)	−0.0015 (5)
C6	0.0146 (6)	0.0182 (6)	0.0147 (6)	0.0009 (5)	0.0015 (5)	0.0012 (5)
C7	0.0129 (6)	0.0155 (6)	0.0136 (6)	−0.0012 (5)	0.0004 (5)	−0.0014 (5)
C8	0.0151 (6)	0.0167 (6)	0.0135 (5)	0.0008 (5)	0.0037 (5)	−0.0014 (4)
C9	0.0167 (6)	0.0169 (6)	0.0169 (6)	−0.0023 (5)	0.0035 (5)	−0.0033 (5)
C10	0.0204 (7)	0.0118 (6)	0.0226 (6)	−0.0015 (5)	0.0071 (5)	−0.0006 (5)
C11	0.0171 (7)	0.0161 (6)	0.0174 (6)	0.0016 (5)	0.0041 (5)	0.0015 (5)
C12	0.0142 (6)	0.0156 (6)	0.0137 (6)	0.0014 (5)	0.0036 (5)	−0.0001 (5)
C13	0.0175 (7)	0.0174 (6)	0.0168 (6)	0.0005 (5)	0.0040 (5)	0.0006 (5)
C14	0.0164 (6)	0.0164 (6)	0.0162 (6)	0.0000 (5)	0.0050 (5)	−0.0012 (5)
C15	0.0168 (7)	0.0169 (6)	0.0189 (6)	−0.0015 (5)	0.0082 (5)	0.0010 (5)
C16	0.0182 (7)	0.0201 (6)	0.0231 (7)	0.0035 (6)	0.0058 (5)	0.0006 (5)
C17	0.0174 (7)	0.0270 (8)	0.0206 (6)	0.0006 (5)	0.0021 (5)	0.0029 (5)
C18	0.0202 (7)	0.0243 (7)	0.0155 (6)	−0.0022 (5)	0.0045 (5)	−0.0007 (5)
C19	0.0183 (6)	0.0171 (6)	0.0172 (6)	−0.0034 (5)	0.0073 (5)	−0.0010 (5)
C20	0.0184 (7)	0.0165 (6)	0.0174 (6)	−0.0035 (5)	0.0071 (5)	−0.0014 (5)
C21	0.0199 (7)	0.0232 (7)	0.0150 (6)	−0.0038 (5)	0.0028 (5)	−0.0018 (5)
C22	0.0172 (7)	0.0251 (7)	0.0219 (7)	−0.0007 (6)	0.0020 (5)	0.0037 (5)
C23	0.0185 (7)	0.0214 (7)	0.0245 (7)	0.0020 (5)	0.0061 (5)	−0.0004 (5)
C24	0.0173 (7)	0.0188 (6)	0.0194 (6)	−0.0038 (5)	0.0082 (5)	−0.0016 (5)
O9	0.0590 (8)	0.0185 (5)	0.0210 (5)	0.0045 (5)	0.0017 (5)	−0.0002 (4)

### Geometric parameters (Å, °)

**Table:**

Zn1—O1	2.1725 (10)	N8—H8m	0.895 (15)
Zn1—O3	2.1886 (11)	C1—C2	1.3868 (18)
Zn1—O5	2.2595 (9)	C1—C6	1.5216 (19)
Zn1—O7	2.2372 (8)	C2—C3	1.390 (2)
Zn1—N1	2.0107 (11)	C2—H2	0.96
Zn1—N2	2.0001 (11)	C3—C4	1.394 (2)
O1—C6	1.2676 (16)	C3—H3	0.96
O2—C6	1.2418 (17)	C4—C5	1.3810 (18)
O3—C7	1.2671 (16)	C4—H4	0.96
O4—C7	1.2460 (16)	C5—C7	1.5256 (18)
O5—C13	1.2607 (16)	C8—C9	1.3832 (18)
O6—C13	1.2481 (15)	C8—C13	1.5163 (17)
O7—C14	1.2577 (15)	C9—C10	1.3871 (17)
O8—C14	1.2508 (14)	C9—H9	0.96
N1—C1	1.3364 (18)	C10—C11	1.3920 (17)
N1—C5	1.3383 (18)	C10—H10	0.96
N2—C8	1.3380 (15)	C11—C12	1.3855 (18)
N2—C12	1.3370 (15)	C11—H11	0.96
N3—C15	1.3604 (18)	C12—C14	1.5240 (16)
N3—C19	1.3672 (16)	C15—C16	1.3945 (17)
N3—H3n	0.854 (13)	C16—C17	1.3846 (19)
N4—C15	1.3413 (17)	C16—H16	0.96
N4—H4n	0.845 (17)	C17—C18	1.378 (2)
N4—H4m	0.838 (14)	C17—H17	0.96
N5—C19	1.3457 (18)	C18—C19	1.3912 (17)
N5—H5n	0.878 (15)	C18—H18	0.96
N5—H5m	0.872 (15)	C20—C21	1.3920 (17)
N6—C20	1.3581 (16)	C21—C22	1.381 (2)
N6—C24	1.3536 (18)	C21—H21	0.96
N6—H6n	0.884 (14)	C22—C23	1.390 (2)
N7—C20	1.3429 (18)	C22—H22	0.96
N7—H7n	0.866 (15)	C23—C24	1.3934 (17)
N7—H7m	0.850 (14)	C23—H23	0.96
N8—C24	1.3437 (17)	O9—H9o	0.83 (2)
N8—H8n	0.840 (18)	O9—H9p	0.866 (19)
			
O1—Zn1—O3	153.16 (4)	O1—C6—O2	126.67 (13)
O1—Zn1—O5	88.95 (4)	O1—C6—C1	115.43 (12)
O1—Zn1—O7	97.34 (4)	O2—C6—C1	117.90 (11)
O1—Zn1—N1	76.69 (4)	O3—C7—O4	127.03 (12)
O1—Zn1—N2	107.41 (4)	O3—C7—C5	115.34 (11)
O3—Zn1—O5	96.92 (4)	O4—C7—C5	117.62 (11)
O3—Zn1—O7	89.46 (3)	N2—C8—C9	121.25 (11)
O3—Zn1—N1	76.46 (4)	N2—C8—C13	114.18 (11)
O3—Zn1—N2	99.43 (4)	C9—C8—C13	124.49 (10)
O5—Zn1—O7	152.52 (3)	C8—C9—C10	118.50 (11)
O5—Zn1—N1	102.81 (4)	C8—C9—H9	120.7524
O5—Zn1—N2	76.41 (4)	C10—C9—H9	120.7525
O7—Zn1—N1	104.67 (3)	C9—C10—C11	119.81 (12)
O7—Zn1—N2	76.18 (3)	C9—C10—H10	120.0969
N1—Zn1—N2	175.74 (5)	C11—C10—H10	120.0967
Zn1—O1—C6	114.92 (9)	C10—C11—C12	118.43 (11)
Zn1—O3—C7	114.97 (9)	C10—C11—H11	120.7863
Zn1—O5—C13	111.69 (7)	C12—C11—H11	120.7859
Zn1—O7—C14	112.33 (7)	N2—C12—C11	121.11 (10)
Zn1—N1—C1	119.10 (9)	N2—C12—C14	113.48 (11)
Zn1—N1—C5	119.51 (9)	C11—C12—C14	125.41 (10)
C1—N1—C5	121.36 (11)	O5—C13—O6	127.57 (11)
Zn1—N2—C8	119.46 (8)	O5—C13—C8	116.49 (10)
Zn1—N2—C12	119.26 (8)	O6—C13—C8	115.91 (11)
C8—N2—C12	120.83 (11)	O7—C14—O8	126.50 (11)
C15—N3—C19	123.53 (10)	O7—C14—C12	116.50 (10)
C15—N3—H3n	119.1 (11)	O8—C14—C12	116.99 (11)
C19—N3—H3n	117.4 (11)	N3—C15—N4	117.86 (11)
C15—N4—H4n	118.0 (10)	N3—C15—C16	118.72 (12)
C15—N4—H4m	121.9 (12)	N4—C15—C16	123.41 (13)
H4n—N4—H4m	120.0 (16)	C15—C16—C17	118.47 (13)
C19—N5—H5n	113.8 (11)	C15—C16—H16	120.7643
C19—N5—H5m	118.9 (12)	C17—C16—H16	120.7632
H5n—N5—H5m	121.5 (15)	C16—C17—C18	121.93 (12)
C20—N6—C24	124.03 (10)	C16—C17—H17	119.0364
C20—N6—H6n	116.7 (10)	C18—C17—H17	119.0372
C24—N6—H6n	119.3 (10)	C17—C18—C19	119.09 (12)
C20—N7—H7n	119.2 (11)	C17—C18—H18	120.4552
C20—N7—H7m	119.1 (12)	C19—C18—H18	120.4557
H7n—N7—H7m	121.5 (16)	N3—C19—N5	117.91 (10)
C24—N8—H8n	119.2 (10)	N3—C19—C18	118.26 (12)
C24—N8—H8m	116.3 (11)	N5—C19—C18	123.81 (12)
H8n—N8—H8m	122.5 (16)	N6—C20—N7	116.75 (11)
N1—C1—C2	120.95 (12)	N6—C20—C21	118.49 (12)
N1—C1—C6	113.37 (11)	N7—C20—C21	124.76 (12)
C2—C1—C6	125.67 (12)	C20—C21—C22	118.59 (12)
C1—C2—C3	118.25 (13)	C20—C21—H21	120.7048
C1—C2—H2	120.8742	C22—C21—H21	120.7055
C3—C2—H2	120.873	C21—C22—C23	121.98 (12)
C2—C3—C4	120.10 (12)	C21—C22—H22	119.0091
C2—C3—H3	119.9475	C23—C22—H22	119.0085
C4—C3—H3	119.949	C22—C23—C24	118.24 (13)
C3—C4—C5	118.32 (13)	C22—C23—H23	120.8827
C3—C4—H4	120.8394	C24—C23—H23	120.881
C5—C4—H4	120.8387	N6—C24—N8	116.41 (11)
N1—C5—C4	121.00 (12)	N6—C24—C23	118.68 (12)
N1—C5—C7	113.41 (11)	N8—C24—C23	124.90 (13)
C4—C5—C7	125.55 (12)	H9o—O9—H9p	104.4 (19)
			
O3—Zn1—O1—C6	−6.33 (14)	Zn1—N1—C1—C2	178.80 (9)
O5—Zn1—O1—C6	97.02 (9)	Zn1—N1—C1—C6	−2.24 (14)
O7—Zn1—O1—C6	−109.82 (9)	C5—N1—C1—C2	0.87 (19)
N1—Zn1—O1—C6	−6.41 (9)	C5—N1—C1—C6	179.83 (11)
N2—Zn1—O1—C6	172.42 (9)	Zn1—N1—C5—C4	−178.36 (9)
O1—Zn1—O3—C7	−4.88 (14)	Zn1—N1—C5—C7	−0.20 (14)
O5—Zn1—O3—C7	−106.37 (9)	C1—N1—C5—C4	−0.43 (19)
O7—Zn1—O3—C7	100.44 (9)	C1—N1—C5—C7	177.72 (11)
N1—Zn1—O3—C7	−4.80 (9)	Zn1—N2—C8—C9	−172.85 (10)
N2—Zn1—O3—C7	176.33 (9)	Zn1—N2—C8—C13	4.06 (14)
O1—Zn1—O5—C13	98.37 (9)	C12—N2—C8—C9	−0.59 (19)
O3—Zn1—O5—C13	−107.90 (9)	C12—N2—C8—C13	176.32 (11)
O7—Zn1—O5—C13	−5.62 (13)	Zn1—N2—C12—C11	170.43 (10)
N1—Zn1—O5—C13	174.47 (9)	Zn1—N2—C12—C14	−9.58 (14)
N2—Zn1—O5—C13	−9.83 (9)	C8—N2—C12—C11	−1.85 (19)
O1—Zn1—O7—C14	−119.53 (9)	C8—N2—C12—C14	178.14 (11)
O3—Zn1—O7—C14	86.52 (9)	N1—C1—C2—C3	−0.37 (19)
O5—Zn1—O7—C14	−17.54 (13)	C6—C1—C2—C3	−179.19 (12)
N1—Zn1—O7—C14	162.37 (9)	N1—C1—C6—O1	−3.56 (16)
N2—Zn1—O7—C14	−13.33 (8)	N1—C1—C6—O2	176.76 (11)
O1—Zn1—N1—C1	4.42 (9)	C2—C1—C6—O1	175.34 (12)
O1—Zn1—N1—C5	−177.61 (10)	C2—C1—C6—O2	−4.34 (19)
O3—Zn1—N1—C1	−175.54 (10)	C1—C2—C3—C4	−0.54 (19)
O3—Zn1—N1—C5	2.43 (9)	C2—C3—C4—C5	0.96 (19)
O5—Zn1—N1—C1	−81.39 (10)	C3—C4—C5—N1	−0.48 (19)
O5—Zn1—N1—C5	96.58 (10)	C3—C4—C5—C7	−178.40 (12)
O7—Zn1—N1—C1	98.66 (10)	N1—C5—C7—O3	−4.10 (16)
O7—Zn1—N1—C5	−83.38 (10)	N1—C5—C7—O4	177.08 (11)
O1—Zn1—N2—C8	−82.06 (10)	C4—C5—C7—O3	173.95 (12)
O1—Zn1—N2—C12	105.56 (10)	C4—C5—C7—O4	−4.86 (19)
O3—Zn1—N2—C8	97.37 (10)	N2—C8—C9—C10	2.52 (19)
O3—Zn1—N2—C12	−75.02 (10)	C13—C8—C9—C10	−174.06 (12)
O5—Zn1—N2—C8	2.46 (9)	N2—C8—C13—O5	−13.36 (17)
O5—Zn1—N2—C12	−169.93 (10)	N2—C8—C13—O6	168.57 (12)
O7—Zn1—N2—C8	−175.54 (10)	C9—C8—C13—O5	163.43 (13)
O7—Zn1—N2—C12	12.07 (9)	C9—C8—C13—O6	−14.64 (19)
Zn1—O1—C6—O2	−173.25 (11)	C8—C9—C10—C11	−2.1 (2)
Zn1—O1—C6—C1	7.10 (14)	C9—C10—C11—C12	−0.2 (2)
Zn1—O3—C7—O4	−175.28 (11)	C10—C11—C12—N2	2.2 (2)
Zn1—O3—C7—C5	6.04 (14)	C10—C11—C12—C14	−177.76 (12)
Zn1—O5—C13—O6	−167.46 (12)	N2—C12—C14—O7	−3.18 (17)
Zn1—O5—C13—C8	14.74 (14)	N2—C12—C14—O8	177.85 (11)
Zn1—O7—C14—O8	−168.70 (11)	C11—C12—C14—O7	176.81 (13)
Zn1—O7—C14—C12	12.44 (14)	C11—C12—C14—O8	−2.2 (2)

### Hydrogen-bond geometry (Å, °)

**Table:**

*D*—H···*A*	*D*—H	H···*A*	*D*···*A*	*D*—H···*A*
C10—H10···O3^i^	0.96	2.49	3.1769 (16)	129
C18—H18···O4	0.96	2.59	3.3556 (16)	137
C22—H22···O3^ii^	0.96	2.59	3.3789 (17)	139
N3—H3n···O8^ii^	0.854 (13)	1.948 (13)	2.7845 (13)	166.0 (16)
N4—H4n···O4^iii^	0.845 (17)	2.096 (17)	2.9287 (16)	168.4 (14)
N4—H4m···O7^ii^	0.838 (14)	2.357 (14)	3.1874 (14)	171.0 (17)
N5—H5n···O4	0.878 (15)	2.206 (14)	3.0600 (14)	164.2 (15)
N5—H5m···O9^iv^	0.872 (15)	2.066 (15)	2.9203 (15)	166.3 (17)
N6—H6n···O6^v^	0.884 (14)	1.763 (14)	2.6381 (13)	169.9 (15)
N7—H7n···O2^vi^	0.866 (15)	2.032 (15)	2.8818 (15)	166.8 (17)
N8—H8n···O2^vii^	0.840 (18)	2.085 (18)	2.9206 (17)	172.4 (17)
O9—H9o···O1^v^	0.83 (2)	2.06 (2)	2.8936 (14)	174 (2)
O9—H9p···O8^viii^	0.866 (19)	1.928 (19)	2.7462 (14)	157.1 (18)

Symmetry codes: (i) −*x*+1, −*y*, −*z*+1; (ii) −*x*+2, *y*+1/2, −*z*+3/2; (iii) −*x*+1, *y*+1/2, −*z*+3/2; (iv) −*x*+1, *y*−1/2, −*z*+3/2; (v) −*x*+1, −*y*+1, −*z*+1; (vi) *x*, −*y*+3/2, *z*+1/2; (vii) −*x*+2, −*y*+1, −*z*+1; (viii) *x*−1, *y*+1, *z*.
